# Sex-specific immune-brain coupling in hippocampal circuits and Alzheimer’s disease vulnerability

**DOI:** 10.21203/rs.3.rs-9580437/v1

**Published:** 2026-05-04

**Authors:** Dana M. Parker, Emily Yi, Whitney Tang, Alison Bamford, Lisa Taylor, Scotty Simmons, Novelle Meza, Nandita Tuteja, Brenda Avila, Evelyn Chang, Jason R. Bock, Liv McMillan, Soyun Kim, Jenna N. Adams, Elizabeth A. Thomas, Emily G. Jacobs, Michael A. Yassa

**Affiliations:** 1. Center for the Neurobiology of Learning and Memory, University of California, Irvine, CA, USA; 2. Department of Neurobiology and Behavior, University of California Irvine, CA, USA; 3. Institute for Interdisciplinary Salivary Bioscience Research, University of California Irvine; 4. Department of Neurosciences, The Scripps Research Institute, La Jolla, CA, USA; 5. Department of Psychological and Brain Sciences, University of California, Santa Barbara, CA, USA; 6. The Ann S. Bowers Women’s Brain Health Initiative, University of California, USA

**Keywords:** Neuroinflammation, sex differences, cytokines, NODDI, diffusion MRI, white matter, hippocampal circuitry, mnemonic discrimination

## Abstract

Women face twice the lifetime risk of Alzheimer’s disease (AD) compared with men. Inflammatory burden is widely implicated in AD, but whether sex-differentiated vulnerability is determined by cytokine levels or by the brain’s sensitivity to those signals remains unclear. Here, we examined sex-specific associations between peripheral inflammatory markers, medial temporal lobe (MTL) white matter microstructure using NODDI diffusion MRI, and hippocampal-dependent memory in 121 cognitively unimpaired older adults. Women exhibited widespread alterations in MTL white matter microstructure relative to men. In women, peripheral cytokines (TNFα, IL-10) were associated with microstructural variation in hippocampal-prefrontal tracts, whereas these associations were absent in men. An indirect association between TNFα and memory through hippocampal cingulum neurite density was observed only in women. Despite these microstructural and coupling differences, cytokine levels were comparable between sexes, indicating that risk may be better captured by the coupling between peripheral immune signals and circuit integrity than by cytokine levels alone. These findings identify a sex-specific pattern of immune-brain coupling that reframes inflammatory risk in AD as a property of circuit-level sensitivity rather than overall inflammatory burden.

Alzheimer’s disease (AD) disproportionately affects women. Even after accounting for differences in longevity, women face approximately twice the lifetime risk of developing AD compared with men^1^ and follow a more aggressive disease course, characterized by accelerated accumulation of pathology, steeper cognitive decline, and greater functional loss^2,3^. Critically, these disparities are not fully explained by survival bias or known demographic risk factors, suggesting that sex-specific *biological mechanisms* contribute to differential vulnerability. Yet, despite decades of research, the upstream processes that render women more susceptible to neurodegeneration remain poorly defined.

Inflammation represents a plausible candidate mechanism for this disparity. Chronic inflammation has been implicated as both a marker and driver of AD risk, with inflammatory-related conditions such as obesity, stroke, and sepsis associated with increased incidence of AD^4^. Importantly, immune processes are robustly sex-dimorphic across the lifespan, with females generally exhibiting heightened immune responsiveness from early adulthood onward^5–7^. Aging in women is marked by a pronounced shift towards low-grade chronic inflammation that is amplified by the menopausal transition^8,9^. This transition involves profound endocrine and immune remodeling, with sustained elevations in pro-inflammatory cytokines, including IL-6, IL-1β, and TNFα, alongside shifts in regulatory mediators such as IL-10^5,10^. These circulating cytokines are not peripherally confined. They can promote blood-brain barrier dysfunction and glial reactivity, providing a mechanistic bridge between peripheral immune aging and central neuroinflammatory activation^5^.

A central assumption in this literature is that elevated inflammatory burden confers risk. However, this framework does not account for whether equivalent levels of circulating cytokines exert similar effects on neural circuits across individuals or sexes. An alternative possibility is that vulnerability is determined by the degree to which peripheral immune signals couple to the integrity of AD-relevant brain circuits. Consistent with this account, microglia (which both produce and respond to TNFα) exhibit sex-specific activation profiles, with female hippocampal microglia showing heightened TNFα-related signaling in the context of aging. This suggests that vulnerability in women may reflect *greater neural sensitivity* to inflammatory signals rather than greater inflammatory exposure ^11^. Despite this evidence, whether sex differences in peripheral inflammatory signaling translate into differential vulnerability of AD-relevant neural circuits, and whether this coupling is detectable before the onset of clinical symptoms, remains unknown.

Medial temporal lobe (MTL) white matter pathways, including the hippocampal cingulum, fornix, and uncinate fasciculus, are among the earliest structures disrupted in AD, which may lead to early deficits in hippocampal-dependent memory. Microstructural changes to white matter in these tracts assessed by diffusion tensor imaging (DTI) have been shown in the context of cognitive decline^12^. However, more advanced multishell diffusion imaging methods, such as neurite orientation dispersion and density imaging (NODDI), can now resolve tissue compartments into biologically interpretable metrics that are capable of detecting more subtle changes that precede overt cognitive decline^13–15^. Prior work has shown that NODDI outperforms traditional DTI in sensitivity to detecting sex differences^16^ and AD-related microstructural decline^17^. Further, converging evidence demonstrates that NODDI measures are sensitive to inflammation-related changes both in humans^18,19^ and rodents^20^, positioning NODDI as particularly well-suited to characterize sex differences in inflammation-related white matter vulnerability and relationships to subtle memory deficits in the preclinical phase of AD.

Characterizing the functional impact of this microstructural decline requires cognitive assessments specifically tuned to medial temporal integrity. Changes in hippocampal-dependent memory emerge prior to clinical symptom onset and can be quantified with sensitive tools, such as the Mnemonic Discrimination Task (MDT)^21,22^. As a paradigm sensitive to hippocampal pattern separation, the MDT detects subtle decline in hippocampal integrity as early as the fourth decade of life and captures variance in hippocampal circuit function that standard neuropsychological batteries miss entirely ^23^. By pairing the MDT with advanced microstructural imaging and peripheral inflammatory profiling, we can effectively test how pathways linking immune signaling to circuit-level dysfunction may confer cognitive deficits in a sex-specific manner.

Here, we tested the hypothesis that AD vulnerability is better captured by *inflammatory sensitivity*, defined as the coupling between peripheral cytokine signaling and MTL circuit integrity, rather than by cytokine levels alone, and that this sensitivity differs by sex. We addressed this question using the Biomarker Exploration in Aging, Cognition, and Neurodegeneration (BEACoN) study sample, a deeply phenotyped cohort of cognitively unimpaired older adults combining advanced multimodal neuroimaging, peripheral cytokine profiling, and cognitive assessment with the hippocampus-dependent MDT. We identify a sex-specific pattern of immune-brain coupling that may serve as a candidate pathway for differential AD vulnerability.

## Results

### Characterization of the Cohort

To investigate the relationships between peripheral inflammation, white matter integrity, and memory, we utilized a well-characterized cohort of N=121 cognitively unimpaired older adults (mean age 69.3 years; 64% female) from the BEACoN study (Table 1). This cohort was specifically selected for its deep phenotyping, including high-resolution multimodal MRI and validated digital cognitive assessments, which have previously been shown to index preclinical AD pathology^22,24–26^. All participants were confirmed to be free of dementia (Clinical Dementia Rating - CDR=0) as well as other major neurological or psychiatric comorbidities. All female participants were postmenopausal. Detailed recruitment criteria are provided in the Methods.

### Sex Differences in MTL White Matter Microstructure

**Figure 1.**
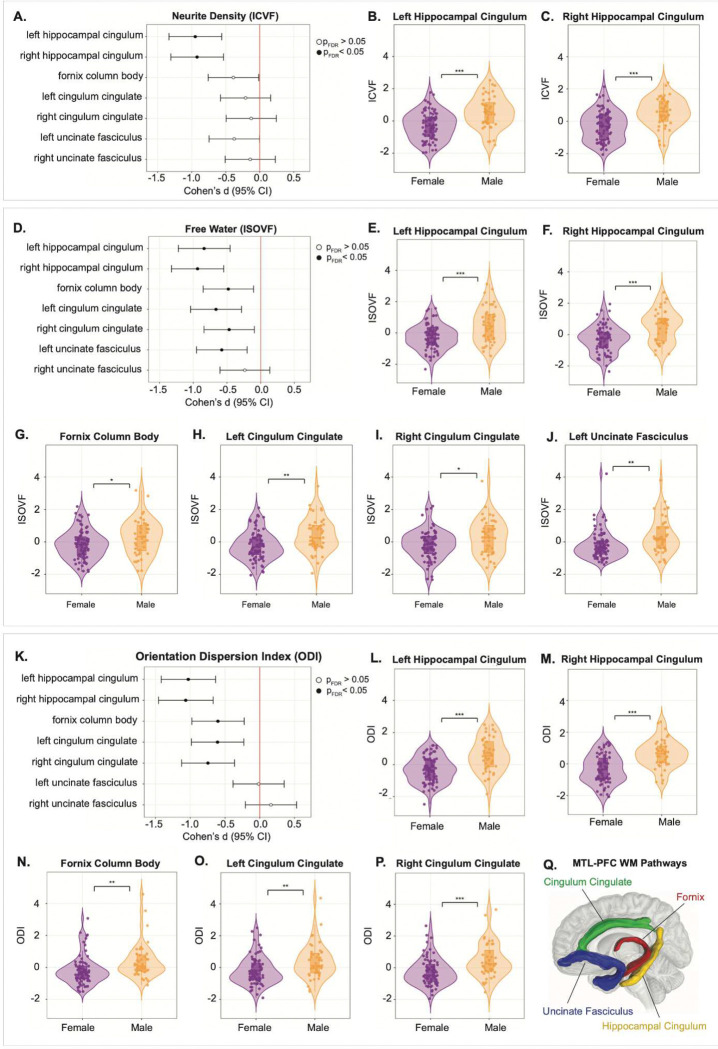
Sex differences in MTL white matter microstructure. Forest plots (**A,D,K**) show effect sizes (Cohen’s d) for sex differences in NODDI-derived diffusion metrics across MTL white matter pathways, with 95% confidence intervals. Results are shown separately for ICVF (**A**), ISOVF (**D**) and OD (**K**). Filled circles indicate regions surviving within-family FDR correction (q < 0.05). The vertical red line denotes no sex difference (d = 0); negative values reflect lower metric values in females relative to males. Violin plots (**B–C,E–J,L–P**) show the distribution of diffusion metrics by sex for selected regions; points represent individual observations and distributions indicate group density. **Q.** Conceptual schematic of MTL-frontal white matter pathways investigated. Significance annotations (*P < 0.05, **P < 0.01, ***P < 0.001) correspond to group comparisons. ICVF, intracellular volume fraction; ISOVF, isotropic volume fraction; ODI, orientation dispersion index.

To test our hypothesis that MTL white matter microstructure is differentially vulnerable in women compared with men, we performed FDR-corrected Wilcoxon rank-sum tests on NODDI diffusion metrics of the hippocampal cingulum, cingulum cingulate, fornix column body, and uncinate fasciculus. Specifically, we examined intracellular volume fraction (ICVF), which serves as a proxy for neurite density, isotropic volume fraction (ISOVF), which reflects extracellular free water and has been associated with neuroinflammatory processes, and orientation dispersion index (ODI), which captures the degree of variability in fiber orientation within a voxel^14^. Our analyses revealed pronounced sex differences in MTL white matter microstructure across multiple NODDI derived metrics (Figure 1A). Women exhibited significantly lower ICVF than men across the hippocampal cingulum (left: Cohen’s d = −0.948, p_FDR_ < 0.001; right: Cohen’s d = −0.921, p_FDR_ < 0.001 ) (Figure 1B-C), indicating reduced neurite density in these tracts compared with men. In contrast, no significant sex differences in ICVF were observed within other MTL areas, suggesting regional specificity to hippocampal-associated tracts, rather than a global white matter effect (Figure 1A).

ISOVF was significantly lower in women across multiple MTL tracts (Figure 1D), including the hippocampal cingulum (left: Cohen’s d = −0.839, p_FDR_ < 0.001; right: Cohen’s d = −0.936, p_FDR_ < 0.001 ) (Figure 1E-F), fornix column body (Cohen’s d = −0.480, p_FDR_ = 0.02) (Figure 1G), cingulum cingulate (left: Cohen’s d =−0.663, p_FDR_ = 0.002; right: Cohen’s d = −0.468, p_FDR_ = 0.02) (Figure 1H-I) and uncinate fasciculus (left: Cohen’s d = −0.578; p_FDR_ = 0.008) (Figure 1J), indicating lower extracellular free water signal. ODI was also lower in women across multiple pathways (Figure 1K), including the hippocampal cingulum (left: Cohen’s d = −1.029, p_FDR_ < 0.001; right: Cohen’s d = −1.065; p_FDR_ < 0.001) (Figure 1L-M), fornix column body (Cohen’s d = −0.602, p_FDR_ = 0.004) (Figure 1N), and cingulum cingulate (left: Cohen’s d = −0.608, p_FDR_ = 0.004; right: Cohen’s d = −0.746, p_FDR_ < 0.001) (Figure 1O-P). MTL pathways are schematically represented in Figure 1Q for reference. Together, these findings demonstrate pronounced and regionally-specific sex differences in MTL white matter microstructure. These structural differences raised the question of whether peripheral inflammatory signaling might differentially couple to white matter integrity in women and men, and whether such coupling could explain the pattern of microstructural vulnerability observed here.

### Coupling between Peripheral Inflammation and MTL White Matter Microstructure is Sex-Dependent

We next tested our hypothesis that peripheral inflammatory markers associate differentially with white matter integrity in women and men. We approached this in two steps: first characterizing the landscape of inflammation-microstructure associations using sex-stratified correlations, then formally testing sex moderation using multiple linear regression models with interaction between sexes and microstructure prediction of measures of inflammation.

#### Correlation analyses reveal selective inflammation-microstructure coupling in women

**Figure 2.**
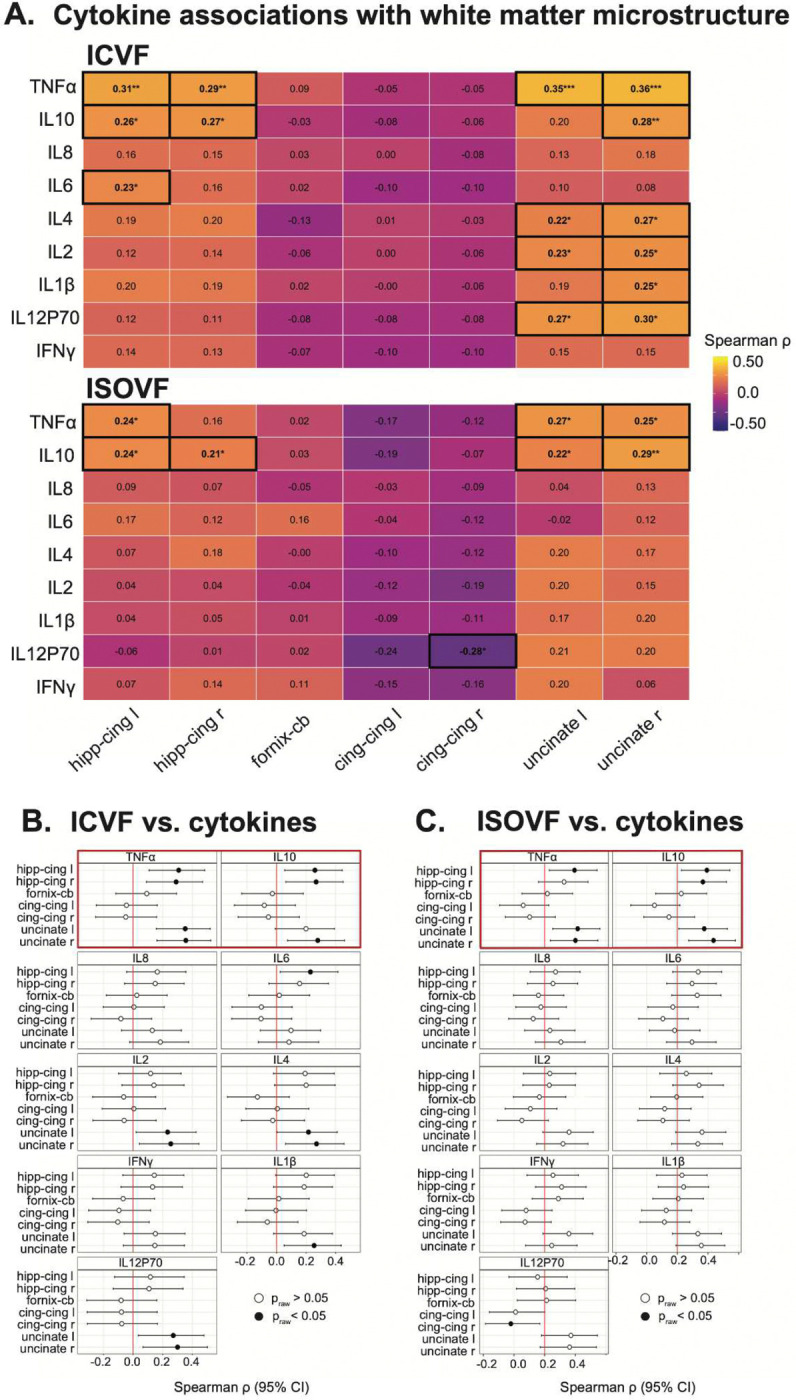
Associations between inflammatory markers and MTL white matter microstructure in females. (**A**) Heatmaps show Spearman correlations (ρ) between log-transformed cytokine concentrations and NODDI-derived diffusion metrics across MTL white matter pathways in females. Top and bottom panels show correlations with ICVF and ISOVF, respectively. Cell color represents the magnitude and direction of associations, with warmer colors indicating stronger positive correlations; numeric values indicate Spearman ρ. Asterisks denote uncorrected significance (*P < 0.05, **P < 0.01, ***P < 0.001), and black outlines highlight significant correlations. (**B,C**) Forest plots show cytokine–diffusion correlations for ICVF (**B**) and ISOVF (**C**), with points representing Spearman ρ and whiskers indicating 95% confidence intervals. Filled circles indicate significant associations (*P < 0.05), and open circles indicate non-significant associations. The vertical red line denotes no association (ρ = 0). Hipp-cing L, hippocampal cingulum left; Hipp-cing R, hippocampal cingulum right; fornix-CB, fornix column/body; cing-cing L, cingulum cingulate left; cing-cing R, cingulum cingulate right; uncinate L, uncinate left; uncinate R, uncinate right; IL, interleukin; TNFα, tumor necrosis factor-α; IFNγ, interferon-γ.

**Figure 3.**
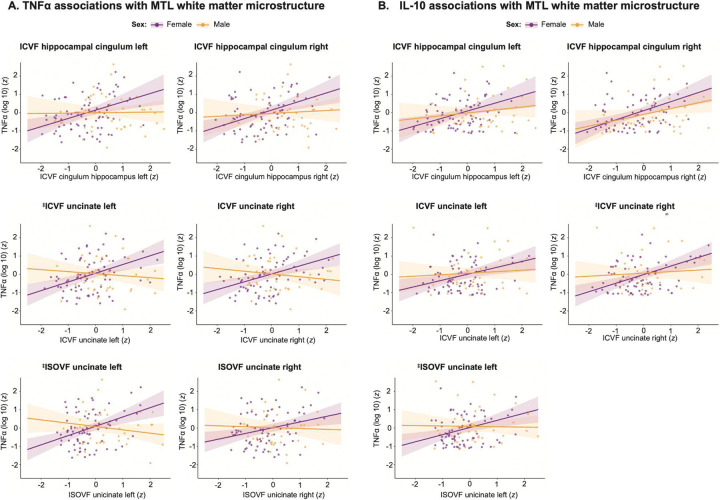
Sex moderation of cytokine-MTL microstructure associations in MTL pathways. (**A,B**) Age-adjusted scatterplots show associations between peripheral cytokine concentrations and NODDI-derived diffusion metrics. Panel **A** shows associations with TNFα and panel **B** shows associations with IL-10. Points represent individual participants. Solid lines indicate sex-specific regression slopes for females (purple) and males (orange), with shaded bands representing 95% confidence intervals. Panels show associations within the hippocampal cingulum and uncinate fasciculus bilaterally for ICVF and ISOVF. Asterisks denote significant cytokine × sex interaction effects (*P < 0.05, **P < 0.01, ***P < 0.001). ^‡^ indicates regions showing a significant main effect of sex. TNFα, tumor necrosis factor-α; IL-10, interleukin-10; ICVF, intracellular volume fraction; ISOVF, isotropic volume fraction; MTL, medial temporal lobe.

Spearman rank correlations computed within sex-stratified samples revealed anatomically specific associations between inflammatory markers and MTL white matter microstructure in women (Figure 2). We note that these correlation analyses were used as discovery/screening analyses; confirmatory moderation models are shown in Figure 3.

In women, TNFα showed the strongest and most consistent associations with MTL NODDI metrics, with higher TNFα positively correlated with ICVF in the bilateral hippocampal cingulum and uncinate fasciculus (Spearman ρ ≈ 0.29 – 0.36, p < 0.01) (Figure 2A, 2B). Parallel associations were observed for ISOVF, with significant positive correlations between TNFα and ISOVF in the uncinate fasciculus (ρ ≈ 0.25 – 0.27, p < 0.05) (Figure 2A, 2C). IL-10 showed a convergent pattern with TNFα, with higher concentrations of IL-10 positively correlated with ICVF in the bilateral hippocampal cingulum and uncinate fasciculus (ρ ≈ 0.26 – 0.28, p < 0.05) (Figure 2A, 2B) and with ISOVF in the right uncinate fasciculus (ρ ≈ 0.29, p < 0.01) (Figure 2A, 2C). The observation of both a pro-inflammatory marker (TNFα) and an anti-inflammatory marker (IL-10) positively associated with white matter integrity in women, but not men, suggests heightened inflammatory sensitivity of MTL white matter in women.

**Extended Data Fig. 1:**
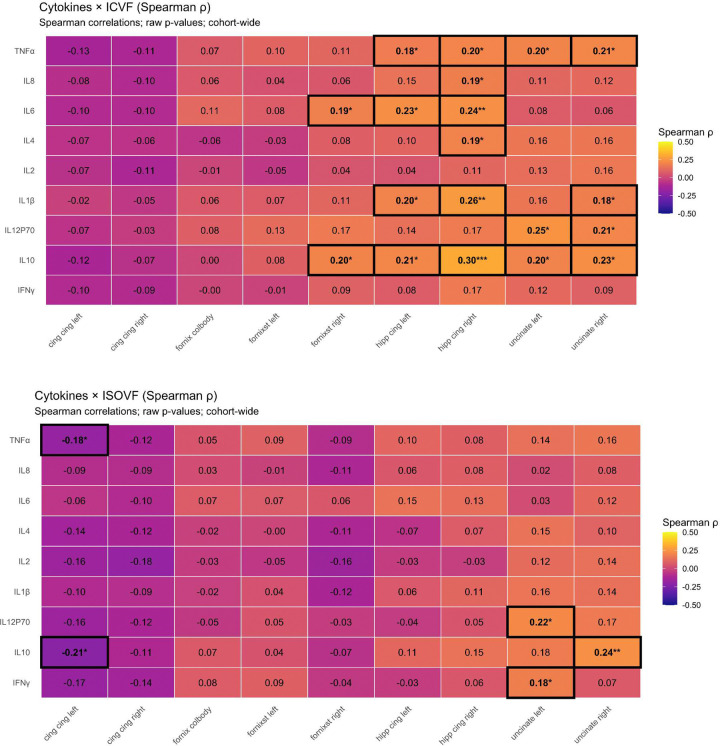
Cohort wide correlogram of inflammatory markers and MTL white matter microstructure. Spearman correlation matrices showing associations between cytokines and NODDI diffusion metrics across MTL white matter pathways in the full cohort. The top panel displays correlations with ICVF, and the bottom panel displays correlations with ISOVF. Cell values represent Spearman ρ, with asterisks indicating raw p-values (*p < 0.05, **p < 0.01, ***p < 0.001; significant associations are additionally outlined. Compared with the female-specific analyses presented in the main text, cohort wide relationships were weaker and less spatially consistent across regions.

**Extended Data Fig. 2:**
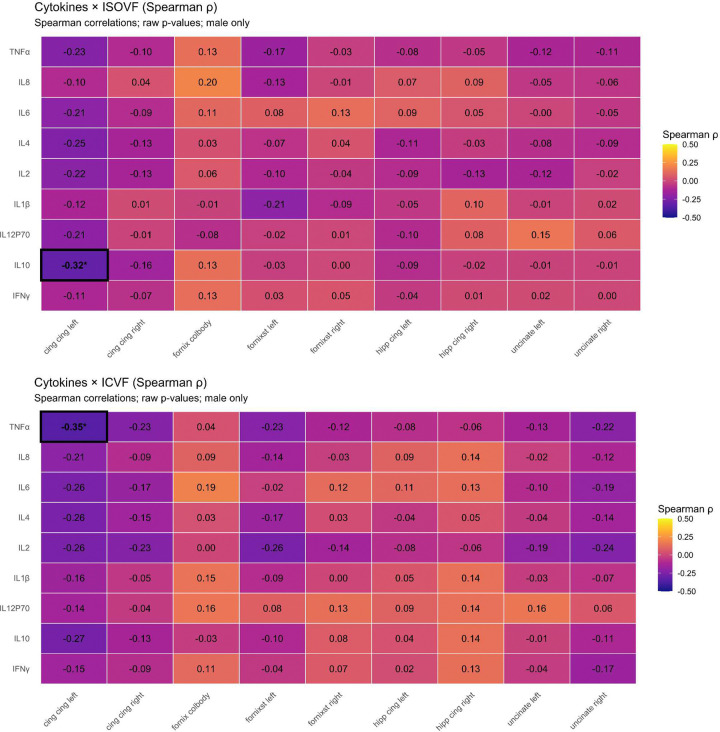
Men only correlogram of inflammatory markers and MTL white matter microstructure. Spearman correlations matrices showing associations between cytokines and NODDI diffusion metrics across the MTL white matter pathways in men only. The top panel displaces correlation with ICVF and the bottom displays correlations with ISOVF. Cell values represent Spearman ρ, with asterisks indicating raw p-values (*p < 0.05) and significant associations outlined. Overall, men exhibited few significant relationships between inflammatory markers and diffusion metrics, with only isolated negative associations observed in the cingulum, indicating weaker and less spatially consistent inflammation microstructure coupling compared with females.

Moderate associations were additionally observed for IL-6 and IL-4 in hippocampal cingulum and fornix segments (ρ ≈ 0.20 – 0.23) (Figure 2A), though these were smaller in magnitude and less spatially consistent. In contrast, corresponding analyses in men revealed weak and spatially inconsistent relationships, with no cytokine showing reproducible associations with MTL white matter metrics (Extended Data Figure 1,2).

As a sensitivity analysis to demonstrate regional specificity, inflammatory associations with white matter integrity in women were *absent* in the cingulum cingulate, an extension of the cingulum bundle outside the MTL proper, suggesting that inflammatory coupling is preferentially concentrated in hippocampal circuits rather than reflecting a global effect of peripheral inflammation on white matter integrity.

#### Sex differences in circulating cytokine levels do not account for microstructural differences

**Extended Data Fig. 3:**
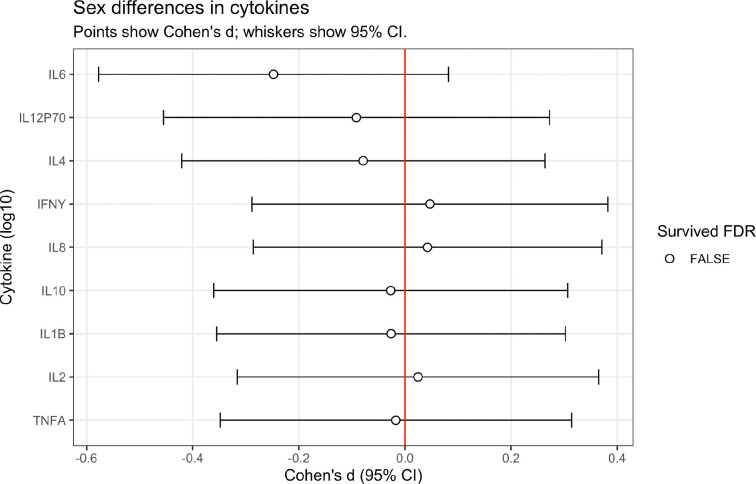
Sex differences in circulating cytokine concentrations. Forest plot displaying effect sizes for sex differences in log-transformed cytokine concentrations. Points represent Cohen’s d for the difference between females and males, and whiskers indicate 95% confidence intervals. The vertical red line denotes no sex difference (d=0). None of the cytokines survived within family FDR correction.

To determine whether the observed sex differences in associations between white matter integrity and peripheral inflammation could be attributed to elevated systemic inflammation in women, we examined sex differences in salivary cytokine concentrations. Wilcoxon rank-sum tests revealed no significant sex differences in any of the measured cytokines, including TNFα and IL-10, following FDR correction for multiple comparisons (Extended Data Fig. 3). Effect sizes for sex differences across the cytokine panel were small, with 95% confidence intervals for Cohen’s *d* inclusive of zero in all cases. Notably, TNFα and IL-10, the markers most strongly implicated in the previous analyses, showed no group-level sex differences. These findings indicate that women and men in this sample were exposed to comparable levels of circulating inflammatory cytokines, suggesting that the sex-dependent associations between inflammation and white matter microstructure cannot be attributed to differences in mean inflammatory burden between sexes.

#### Regression analyses formally confirm sex moderation of inflammation-microstructure associations

To test whether the sex differences observed in correlational analyses reflected statistically reliable moderation of inflammation-microstructure associations by sex, beyond mean-level differences in cytokines or white matter metrics, we fit age-adjusted linear regression models incorporating cytokine × sex interaction terms.

TNFα demonstrated strong sex-dependent associations with MTL white matter. In women, higher ICVF was strongly and positively associated with TNFα in the bilateral hippocampal cingulum (left: β = 2.20, SE = 0.68, p = 0.0017; right: β = 2.33, SE = 0.66, p = 0.001) and bilateral uncinate fasciculus (left: β = 2.69, SE = 0.66, p < 0.001; right: β = 2.72, SE = 0.71, p < 0.001). Corresponding associations in men were small and non-significant (all p > 0.35). Formal tests of sex moderation confirmed significantly steeper TNFα-NODDI slopes in women than men across these tracts (e.g., uncinate left: Δβ = 3.31, p = 0.0015; uncinate right: Δβ = 3.66, p = 0.0030) (Figure 3A). A parallel pattern was observed for ISOVF, with TNFα significantly associated with uncinate ISOVF in women (left: β = 4.44, SE = 1.08, p < 0.001; right: β = 3.70, SE = 1.25, p = 0.0037) but not men, yielding large sex differences in slope (left uncinate: Δβ = 6.03, p < 0.001) (Figure 3A).

IL-10 showed a similar but attenuated pattern to TNFα. In women, IL-10 was positively associated with ICVF in the hippocampal cingulum (left: β = 1.82, SE = 0.62, p = 0.0039; right: β = 2.19, SE = 0.58, p < 0.001) and uncinate fasciculus (left: β = 1.74, SE = 0.61, p = 0.0049; right: β = 2.60, SE = 0.62, p < 0.001), while associations in men were largely absent (Figure 3B). Sex differences in slope reached significance for right uncinate ICVF (Δβ = 2.13, p = 0.048) and left uncinate ISOVF (Δβ = 3.21, p = 0.029) (Figure 3B). All primary regression results were robust to the exclusion of influential observations identified via Cook’s distance, confirming that findings were not driven by a small number of extreme values.

### Coupling Between MTL White Matter Integrity and Hippocampal-Dependent Memory is Sex-Dependent

Having established that peripheral inflammatory markers couple more strongly to MTL white matter microstructure in women than men, we next examined whether this sex-specific microstructural variation carries functional consequences for hippocampal-dependent memory. We focused on temporal mnemonic discrimination performance (MDT-T) as our primary cognitive outcome given its established sensitivity to hippocampal-prefrontal circuit integrity^27^ and preclinical AD-related change^28^.

#### Sex moderates the relationship between MTL white matter integrity and memory

**Figure 4.**
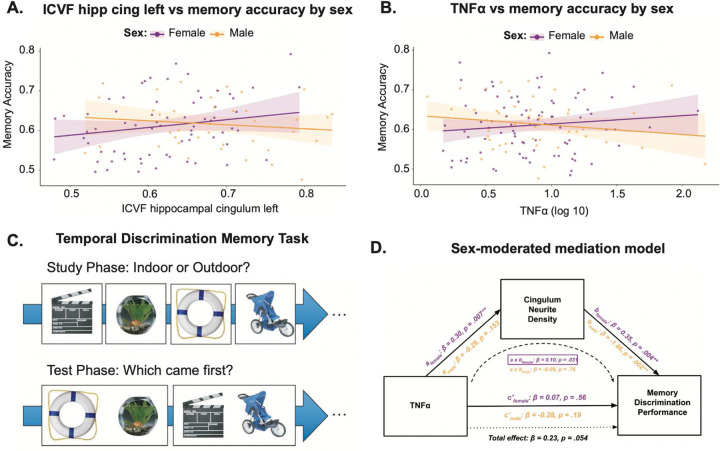
Sex-stratified and sex-moderated associations between inflammation, white matter microstructure, and temporal discrimination memory. (**A,B**) Age-adjusted scatterplots show sex-specific associations between hippocampal cingulum ICVF and temporal discrimination memory accuracy (**A**), and between and peripheral TNFα levels and temporal discrimination memory accuracy (**B**). Points represent individual participants. Solid lines indicate age-adjusted linear fits within each sex (female, purple; male, orange), with shaded bands representing 95% confidence intervals. (**C**) Temporal discrimination memory task schematic showing the two phases of the procedure. (**D**) Sex-moderated mediation model testing whether hippocampal cingulum ICVF mediates the relationship between TNFα and mnemonic discrimination performance, with sex moderating model pathways. Values shown are standardized regression coefficients. TNFα, tumor necrosis factor-α; ICVF, intracellular volume fraction.

Regression models revealed a significant interaction between left hippocampal cingulum ICVF and sex in predicting MDT-T performance (β = −0.72, p = 0.04) (Figure 4A), indicating that the microstructure-memory relationship differed by sex. Consistent with this interaction, sex-stratified models showed a marginal positive association between hippocampal cingulum ICVF and performance in women (β = 0.46, p = 0.06) (Figure 4A) that was absent in men. This pattern is interpretable within the context of the moderated mediation framework described below. A trend-level interaction between TNFα and sex predicting performance was also observed (β = −0.10, p = 0.09) (Figure 4B), consistent with the broader pattern of sex-specific inflammatory coupling and motivating formal examination of the mediated pathway. The design of the MDT-T task is shown in Figure 4C and described in the Methods section.

#### Hippocampal white matter mediates the association between TNFα and memory selectively in women

To formally test whether hippocampal cingulum ICVF mediates the relationship between TNFα and memory in a sex-dependent manner, we conducted a moderated mediation analysis (Figure 4D). This framework is appropriate for evaluating conditional indirect effects when the pathway from predictor to outcome is hypothesized to operate through a mediator whose relevance differs by group, which is precisely the architecture our prior findings suggest.

Higher TNFα significantly predicted higher hippocampal cingulum ICVF in women (path a: β = 0.30, p = 0.007), and this relationship was not significant in men (β = −0.29, p = 0.15). Higher hippocampal cingulum ICVF predicted better memory in women (path b: β = 0.35, p = 0.0004), and worse memory in men (β = −1.86, p = 0.002), indicating that the functional relevance of white matter microstructure for memory differed by sex. The indirect effect of TNFα on memory through hippocampal cingulum ICVF was significant in women (β = 0.10, p = 0.031) and absent in men (β = −0.01, p = 0.76), and the sex difference in indirect effects was statistically reliable (β = −0.11, p = 0.024). The direct effect of TNFα on memory was not significant in women (β = 0.07, p = 0.56), nor men (β = −0.28, p = 0.19), indicating that the association between TNFα and memory in women was statistically consistent with an indirect pathway through white matter microstructure (Figure 4D).

## Discussion

Here, we report a pattern of immune-brain coupling that reframes sex-specific inflammatory risk for AD as a property of circuit-level sensitivity rather than inflammatory burden. MTL white matter microstructural features were differentiated by sex, wherein women had lower mean ICVF and ISOVF than men. Peripheral inflammatory markers were differentially associated with MTL white matter microstructure by sex, with women exhibiting stronger and more anatomically consistent associations across cytokines and tracts. This indicates that sex differences in mean microstructure and within-sex immune–brain coupling reflect distinct effects. Rather than explaining the lower group-level microstructural values in women, peripheral cytokines appeared to track individual differences in MTL tissue state among women. The parallel positive associations with ICVF and ISOVF suggest a regulated immune–tissue coupling process involving both neurite density and extracellular water compartments, rather than a simple model in which higher inflammatory burden uniformly predicts microstructural injury.

These effects were not explained by differences in circulating cytokine concentrations, indicative of a sex-specific sensitivity to inflammatory signaling. Notably, these associations were concentrated in hippocampal-prefrontal pathways, including the hippocampal cingulum and uncinate fasciculus, and were absent in non-hippocampal white matter tracts (i.e., cingulum cingulate bilaterally). This regional selectivity argues against a global effect of systemic inflammation on white matter, pointing instead to the preferential vulnerability of circuits among the earliest and most consistently disrupted in AD.

Moderated mediation analyses confirmed the functional relevance of this immune-brain coupling, identifying MTL WM microstructure as a sex-specific pathway linking peripheral TNFα to hippocampal-dependent memory. A key feature of this scheme is that while TNFα was associated with hippocampal cingulum integrity comparably across sexes, the cognitive relevance of that microstructural variation was unique to women. Specifically, white matter integrity in hippocampal-prefrontal pathways predicted mnemonic discrimination performance in women but not in men. Consequently, the indirect pathway from peripheral inflammation to memory was significant only in women, suggesting that women’s brains are not merely exposed to a more inflammatory environment, but are *differentially sensitized* to its neural and cognitive consequences.

These findings align with evidence of more pronounced associations between white matter integrity and cognitive performance in women^29^, and identify a specific inflammatory driver of this variability. We have previously shown in the same cohort that the cognitive outcome most sensitive to this pathway, temporal discrimination accuracy, is selectively vulnerable to preclinical amyloid deposition in frontotemporal memory circuits^26^. Thus, the inflammation-white matter-memory chain identified here may represent a functional signature of circuit vulnerability that precedes clinical symptoms of AD.

NODDI-derived metrics revealed regionally specific sex differences in MTL white matter integrity, with women showing lower neurite density, lower extracellular free water signal, and lower fiber dispersion across hippocampal-associated pathways. While traditional DTI has yielded inconsistent findings regarding sex differences in white matter microstructure during aging^30–33^ , the multi-compartment specificity of NODDI provides a more sensitive framework for detecting sex-dimorphic microstructural shifts^16,17^. Our findings suggest that such biophysical modeling reveals a female-specific vulnerability in these critical memory circuits.

These microstructural profiles may presage an accelerated accumulation of AD-related pathological burden in women, consistent with evidence of sex-dimorphic tau propagation and white matter involvement in early disease stages^34^. Alternatively, these differences may inherently amplify circuit vulnerability, compromising the structural integrity of circuits through which pathology propagates. While the cross-sectional nature of these data precludes directionality, our findings suggest that stronger immune-white matter coupling in women may contribute to these differences. We hypothesize that age-related shifts in inflammatory signaling, particularly during the menopausal transition, contribute to the emergence and widening of these microstructural sex differences across the lifespan.

The strong, consistent association between salivary cytokines (TNFα, IL-10) and MTL white matter integrity in women, contrasted with the weak, inconsistent patterns in men, reveals a dimension of sex-specific sensitivity that has been largely overlooked in aging research. While elevated systemic inflammation is traditionally associated with poorer cognitive performance^35,36^ and white matter integrity in aging^37–39^, higher cytokine levels are often linked to tissue compromise in pathological states^40–43^. Yet our findings in cognitively unimpaired adults suggest that elevated TNFα and IL-10 may index adaptive immune surveillance and tissue maintenance. The parallel positive associations of both pro- and anti-inflammatory markers (TNFα and IL-10, respectively)^44,45^ suggest a coordinated immune response that is inconsistent with a simple model of inflammatory toxicity. It is more likely a form of regulated coupling between immune signaling and tissue state, consistent with context-dependent immune-brain interactions described in prior work.

This sex-dependent coupling likely reflects fundamental differences in central immune regulation. At the cellular level, microglia, which both produce and respond to TNFα, exhibit sex-specific activation profiles^46^, with female hippocampal microglia showing heightened TNFα-related signaling in the brain during aging^11^. In healthy aging, this pronounced immune signaling appears to support homeostatic immune-tissue interactions, yet this state represents a double-edged sword. While it maintains circuit integrity under stable conditions, it may paradoxically amplify vulnerability to chronic dysregulation as AD-related pathological burden accumulates. This adaptive-to-maladaptive transition may be further catalyzed by endocrine remodeling during the menopausal transition. Estrogen exerts broad neuroprotective effects by modulating microglial activation and maintaining blood-brain barrier integrity^47^; its withdrawal removes a key buffer against inflammatory dysregulation, potentially “unmasking” latent vulnerabilities in the hippocampal cingulum.

Whether menopausal hormone therapy (MHT) can preserve this buffer remains an essential, untested question. While our sample consisted entirely of postmenopausal women, specific data regarding the initiation, type, and duration of MHT was not available in the current cohort. Given the known heterogeneity in MHT timing and formulation, larger longitudinal studies are required to determine if exogenous estrogen can maintain the adaptive inflammatory sensitivity or delay its pathological transition.

Several considerations bear on the interpretation of these findings. First, the cross-sectional design precludes strong conclusions about the directionality of the observed associations. Mediation models are used here to test conditional statistical architecture rather than temporal causality. Longitudinal studies tracking inflammatory profiles across the menopausal transition are required to establish temporal precedence and evaluate whether the immune-brain coupling identified here predicts subsequent neurodegeneration. Second, while salivary assays have been validated against plasma in this cohort^54^, additional validation against CSF or PET-based assays is a natural future direction. That said, the associations between salivary-based inflammatory markers and circuit-specific outcomes support biological relevance as a scalable peripheral assay. Third, women were more represented in the cohort, consistent with enrollment patterns in aging studies and the epidemiology of AD risk. Robust standard errors and sensitivity analyses were used to account for unequal group sizes. Fourth, salivary cytokines may reflect local oral inflammatory processes. To address this concern, we implemented rigorous quality control to exclude analytes below the LLOD and remove participants with profiles indicative of acute inflammation as extreme outliers. Nevertheless, future replication with plasma and CSF assays will be important. Finally, future work integrating genetic risk factors (e.g., ApoE ε4 status) and co-pathologies (e.g., vascular and metabolic conditions) will be essential to partition the relative contributions of these factors to the sex-specific microstructural profiles observed here.

These results have important implications for how sex differences in neurodegeneration risk are conceptualized^48^. Our data suggest that risk models based solely on circulating cytokine concentrations may overlook a critical dimension of vulnerability. Individuals with comparable peripheral inflammatory profiles can differ markedly in how those signals map onto vulnerable neural circuits. Inflammatory sensitivity may therefore represent a more informative axis of risk than inflammatory burden alone. In parallel, our results support peripheral cytokine profiling as a scalable and minimally invasive approach for indexing the immune-brain interface during the preclinical phase of AD.

More broadly, these findings position the peripheral immune-brain interface as a candidate pathway for sex-specific AD vulnerability that is measurable before the onset of clinical symptoms. Longitudinal studies are now needed to determine whether this coupling predicts subsequent white matter decline, tau accumulation, or cognitive deterioration, and whether it can be modified through immune, hormonal, or lifestyle interventions. By identifying specific circuits through which peripheral biology relates to early neural vulnerability, this work motivates future precision prevention strategies and helps identify women most likely to benefit from early intervention.

## Methods

### Participants and Study Design

Participants were recruited from the Biomarker Exploration in Aging, Cognition, and Neurodegeneration (BEACoN) Study at the University of California, Irvine (NIA R01AG053555; PI: Yassa). This ongoing study is designed to characterize early biological signatures of neurodegeneration risk through deep multimodal phenotyping, including high-resolution structural, functional and diffusion MRI, validated digital cognitive assessments including the MDT, and collection of peripheral biospecimens, enabling comprehensive examination of brain-behavior-immune relationships in aging. Prior work in this cohort has established that plasma and salivary biomarkers reflect AD-relevant pathological burden^24,25^, that performance on the mnemonic discrimination task indexes preclinical amyloid accumulation with diagnostic sensitivity^22,26^ , and that white matter integrity in MTL pathways predicts memory performance^49^, collectively validating the core measures and analytic approach of the present study.

Participants were enrolled if they were 60–85 years old, possessed visual and auditory acuity adequate to complete cognitive assessments, and were cognitively unimpaired as defined by a score of zero on the Clinical Dementia Rating (CDR) scale^50^. Exclusion criteria included a history of significant neurological or psychiatric comorbidity, alcohol or substance use disorder within the preceding two years, major medical conditions with known effects on cognition, or a diagnosis of mild cognitive impairment, dementia, or other cognitive disorder. While all women included were postmenopausal, specific data regarding the initiation, type, and duration of MHT was not available. All experimental protocols were approved by the Institutional Review Board of the University of California, Irvine, and all participants provided written informed consent prior to enrollment.

The final analytic sample for the present study comprised 121 participants (78 female, 43 male; mean age 69.3 years) with complete multi-shell diffusion MRI and salivary cytokine data. Comprehensive demographic and clinical characteristics are detailed above in Table 1.

The ratio of women to men reflects the higher epidemiological burden of AD risk in women and is consistent with the sex distribution of cognitively unimpaired aging cohorts more broadly. To account for this imbalance, all sex-interaction models were evaluated using robust standard errors. Sensitivity analyses and bootstrapping were employed to ensure that the observed sex-specific effects were driven by biological divergence rather than differences in group-level statistical power or variance heteroscedasticity.

### Temporal Mnemonic Discrimination Task

We used the Temporal Mnemonic Discrimination Task as the primary cognitive outcome based on its sensitivity to preclinical amyloid accumulation in frontotemporal circuits^26^, its known dependence on hippocampal-prefrontal cortical processing^51^, and its hypothesized sensitivity to the MTL-prefrontal white matter pathways examined here (i.e., fornix, cingulum, and uncinate fasciculus). During the study phase participants made an incidental judgment in response to pictures of everyday items (“indoor” vs. “outdoor”) presented for 2000 msec each (500 msec inter-stimulus interval). During the test phase they were shown pairs of items that were previously presented during study and made a temporal order judgment (which came first?). We varied similarity/interference by varying the temporal lag between the items that were tested. We focused on the high similarity lure trials (items presented close together in time), which were the more challenging judgments and a more sensitive probe of hippocampal-prefrontal memory. A schematic of the task is shown in Figure 4C.

### Saliva Cytokine Collection and Quantification

Unstimulated whole saliva (~2 ml) was obtained via passive drool between 7am and 5pm according to previously established protocols^52,53^. Participants were asked to refrain from smoking, eating, drinking, or oral hygiene procedures for 30–60 minutes prior to collection. Samples were frozen at −80C within one hour, and subsequently thawed and centrifuged (5000 g; 15 min; 4°C) to remove mucins and cellular debris prior to analysis.

Ten cytokines (IL-1β, IL-2, IL-4, IL-6, IL-8, IL-10, IL-12p70, IL-13, TNFα, and IFN-γ) were measured using the V-PLEX Proinflammatory Cytokine Panel 1 (Meso Scale Discovery, Gaithersburg, MD). This electrochemiluminescence (ECL)-based multiplex immunoassay was selected for its superior dynamic range and sensitivity of detection compared with conventional ELISA. Assays followed manufacturer protocol with minor modifications validated for salivary matrices^54^. To maximize statistical power, salivary samples collected and analyzed in two separate waves (2023 and 2025) were combined. Cross-wave compatibility was verified by log10-transforming concentrations and modeling as a function of collection wave. Notably, IL-13 was excluded from all final analyses due to higher inter-assay variability and poor technical reproducibility across runs.

Concentrations (pg/ml) were determined using MSD Discovery Workbench Software using curve fit models. Lower limits of detection (LLoD) were calculated as the concentration corresponding to the signal 2.5 times standard deviation above background. Across both analytical waves, the mean intra-assay coefficients of variation (CV) were <15% for most analytes. The most conservative, or highest, LLODs were as follows: IL-1β (0.036 pg/ml, 10.5%), IL-2 (0.041 pg/ml, 9.23%), IL-4 (0.03 pg/ml, 7.78%), IL-6 (0.079 pg/ml, 12.81%), IL-8 (0.039 pg/ml, 9.17%), IL-10 (0.052 pg/ml, 9.0%), IL-12p70 (0.011 pg/ml, 16.3%), TNFα (0.05 pg/ml, 10.01%), and IFN-γ (0.028 pg/ml, 4.4%). Concentrations were log10-transformed to address positive skew.

While plasma is traditionally used for systemic inflammatory profiling, salivary cytokine measurement provides a non-invasive, scalable alternative validated within the BEACoN cohort. Our prior work in this cohort^54^ demonstrated significant positive associations between plasma and salivary concentrations for IFNy, IL-6, and TNFα, though absolute concentrations were consistently higher in saliva, likely reflecting the unique immunological environment of the oral mucosa. The broader 10-plex panel was included to allow for discovery-based characterization of the brain-immune relationship^55^. Although direct oral health indicators were unavailable, we implemented a rigorous statistical quality control pipeline to exclude analytes below the LLOD and identify participants with profiles indicative of acute inflammation (i.e. extreme outliers), ensuring the stability of the inflammatory signal.

### MRI Acquisition

All participants were scanned at the University of California, Irvine on a 3T Prisma scanner (Siemens Medical Systems) equipped with a 32-channel head coil. A whole-brain T1-weighted volumetric image was acquired using a magnetization-prepared rapid acquisition gradient echo (MPRAGE) sequence (voxel size: 0.8 mm isotropic; TR: 2300 ms; TE: 2.38 ms; TI: 902 ms; flip angle: 8°). Multi-shell diffusion-weighted images were acquired using a Pulsed Gradient Spin Echo with single shot echo planar imaging (EPI) read out with 72 interleaved axial slices (voxel size: 1.7 mm isotropic; FOV: 218 mm; matrix: 128 × 128; TR: 3500 ms; TE: 102 ms). Three diffusion shells were acquired at b = 0, 1500, and 3000 s/mm^2^, with 64 diffusion directions per shell.

### Structural and Diffusion MRI Processing

T1-weighted images were preprocessed using Freesurfer v7.4^56^, including correction for head motion and intensity inhomogeneity and removal of non-brain tissue. Diffusion scans were preprocessed using QSIPrep v0.24.0^57^, a standardized preprocessing pipeline that integrates tools from FSL, MRtrix3, and ANTs. Raw diffusion images were denoised using MP-PCA^58^ via MRTrix3’s dwidenoise^59^, followed by correction of head motion and eddy current distortions using FSL’s eddy^60^ with model-based motion correction. Preprocessed images were then resampled to 1mm isotropic resolution prior to microstructural modeling.

NODDI microstructural modeling was performed using QSIRecon (v0.24.0^57^) via the Accelerated Microstructure Imaging via Convex Optimization (AMICO) framework^14,15^, incorporating participant-specific anatomical information from FreeSurfer segmentations. Outputs included three NODDI metrics: intracellular volume fraction (ICVF; indexing neurite density and white matter integrity), isotropic volume fraction (ISOVF; indexing extracellular free water fraction, a marker sensitive to neuroinflammatory processes, perivascular edema, and tissue loss), and orientation dispersion index (ODI; capturing the angular complexity of fiber architecture). Microstructural maps were used for subsequent region-of-interest (ROI) based analyses defined using the Johns Hopkins University ICBM-DTI-81 white matter atlas^61^. ROIs were selected a priori based on their relevance to the MTL connectivity and AD vulnerability.

### Statistical Analyses

All analyses were performed in R (4.1.2), including structural equation modeling conducted using the *lavaan* package^62^. Statistical significance was evaluated using two-tailed tests with α = 0.05 throughout, except where noted. Initial correlational analyses were conducted as a screening step to identify candidate cytokine-region associations (Extended Data Fig. 1–2). The identified cytokine-region correlations were then corrected for multiple comparisons using within-family false discovery rate (FDR), with family defined as a given NODDI metric and cytokine pair, across all regions.

Regression and mediation models were then restricted to a limited set of predefined cytokine-regions. Primary analyses adjusted for age to account for biological differences known to influence white matter microstructure. Sex differences in MTL WM microstructure and cytokine concentrations were evaluated using Wilcoxon rank-sum tests with FDR correction, and effect sizes were estimated using Cohen’s d with 95% confidence intervals.

To assess the landscape of inflammation-microstructure associations (discovery), Spearman rank correlations were computed between inflammatory markers and NODDI-derived ICVF and ISOVF within sex-stratified samples. These analyses were used to evaluate the strength, direction, and anatomical specificity of cytokine-tract relationships and to confirm the selection of TNFα and IL-10 as the primary inflammatory markers for subsequent modeling, a selection informed *a priori* by their established relevance to AD pathophysiology and sex-dimorphic immune aging^5,11^. ODI was examined in sex difference analyses but excluded from inflammation-focused modeling given its more complex interpretation in the context of crossing fibers.

Moderation by sex was tested using age-adjusted models with cytokine × sex interaction terms. Significant interactions were followed by sex-stratified simple slope estimation to characterize within-sex associations. Model fit was summarized using adjusted R^2^.

To examine the joint associations among peripheral inflammation, MTL white matter microstructure, and mnemonic discrimination, we conducted *moderated mediation analyses*. Behavioral performance on the more difficult high-similarity trials in the MDT-T was selected as the primary cognitive outcome due to its heightened sensitivity to subtle brain changes. TNFα was the primary inflammatory predictor and hippocampal cingulum ICVF the mediating variable, selected based on their *a priori* relevance to hippocampal-prefrontal connectivity in AD^12^ and confirmed by the strongest and most anatomically consistent associations in correlation and regression analyses. Sex was included as a moderator on all paths in the mediation model (total, direct, and indirect).

In accordance with contemporary statistical frameworks, the significance of the indirect pathway was prioritized as the primary metric of immune-brain-behavior coupling. This approach remains reliable even when individual path components exhibit marginal magnitudes, as the model evaluates the conditional dependence of mnemonic performance on the inflammation-white matter interface rather than isolated indirect effects. The indirect effect of TNFα on MDT-T performance through hippocampal cingulum ICVF was estimated separately within each sex, and the sex difference in indirect effects was formally tested. Sensitivity analyses excluding influential observations identified via Cook’s distance were conducted for all primary regression and mediation models.

## Extended Data

## Figures and Tables

**Table 1.** Demographics.

Variable	Female (n=78)	Male (n=43)	Total (n=121)	p-value
Age, years (Mean [SD])	68.9 [6.7]	70 [6.4]	69.3 [6.6]	0.353
Education, years (Mean [SD])	16.2 [2.4]	17 [2.4]	16.5 [2.4]	0.072
Hispanic ethnicity (n,%)	12,15.4%	6, 14%	18, 15%	1.000^†^
MMSE (Mean [SD])	28.3 [1.6]	28.4 [1.7]	28.4 [1.7]	0.832
ApoE ε4 carrier (n,%)	25, 32.1%	16, 37.2%	41, 34%	0.664^†^
TNFα (Median[IQR])	0.78 [0.55,1.13]	0.83 [0.52,1.13]	0.78 [0.53,1.13]	0.968^‡^
IL-10 (Median[IQR])	0.30 [0.16, 0.58]	0.33 [0.19,0.62]	0.31 [0.16, 0.60]	0.627^‡^

† Chi-square tests for categorical variables;

‡ Wilcoxon rank-sum tests for non-normally distributed variables; all other comparisons used independent samples t-tests. MMSE, Mini-Mental State Examination; ApoE ε4, apolipoprotein E ε4; TNFα, tumor necrosis factor-α; IL-10, interleukin-10.
